# Maternal health care visits as predictors of contraceptive use among childbearing women in a medically underserved state in Nigeria

**DOI:** 10.1186/s41043-018-0150-4

**Published:** 2018-07-24

**Authors:** Anthony Idowu Ajayi, Oladele Vincent Adeniyi, Wilson Akpan

**Affiliations:** 1 Department of Sociology, Faculty of Social Sciences & Humanities, University of Fort Hare, 50 Church street, East London, 5200 South Africa; 2 Department of Family Medicine, Walter Sisulu University, Cecilia Makiwane Hospital, East London Hospital Complex, East London, South Africa

**Keywords:** Family planning, Contraceptive, Modern contraceptive, Health care visits, Nigeria, Knowledge of contraceptives, Medically underserved settings

## Abstract

**Background:**

Health care visits during pregnancy, childbirth and after childbirth may be crucial in expanding the uptake of contraceptive care in resource-poor settings. However, little is known about how health care visits influence the uptake of modern contraception in Nigeria. The focus of this paper was to examine how health care visits influence the use of contraceptives among parous women in a medically underserved setting.

**Methods:**

The study adopted a descriptive survey design. Data was collected from 411 women who gave birth between 2010 and 2015 selected through a two-stage cluster random sampling technique. Health care visits for antenatal care services, childbirth, postnatal care and modern contraceptive were dichotomised (yes, no). Descriptive analyses were performed, and percentages, frequencies and means were reported. Multiple logistic regressions were computed, and odds ratios and 95% confidence intervals were calculated.

**Results:**

Knowledge of all contraceptive methods was lowest among women who reside in rural areas. Health care visits for antenatal care (UOR 4.5; 95% CI 2.0–10.5), childbirth (UOR2.1; 95% CI 1.4–3.2) and postnatal care services (UOR 2.3; 95% CI 1.5–3.5) independently predict ever use of any contraceptive methods. Likewise, health care visits for antenatal care (UOR 5.6; 95% CI 2.1–14.8), childbirth (UOR 2.3; 95% CI 1.5–3.6) and postnatal care services (UOR 2.8; 95% CI 1.8–4.5) were independent predictors of current use of modern contraceptive methods. In the adjusted model, health care visits for antenatal care services (AOR 3.2; 95% CI 1.1–8.8) were significantly associated with the use of modern contraceptive methods.

**Conclusion:**

Health care visits significantly predict the use of modern contraceptive methods. Expanding access to health care services would potentially increase contraceptive use among childbearing women in the medically underserved settings.

## Background

Despite the numerous health benefits of family planning (FP), studies suggest that many women in sub-Saharan Africa are not using any contraceptive methods [1–3]. Consequently, unintended pregnancy and abortion-related mortality are high in sub-Saharan Africa [1, 4]. The literature is replete with reasons for underutilisation of modern contraceptives among women in sub-Saharan Africa [5–7]; nevertheless, there is still some ambiguity regarding how best to expand the uptake of modern contraceptives especially in underserved settings. Studies have shown that women’s level of education and overall socioeconomic status are associated with the use of modern contraception [3, 8]. Thus, focusing interventions on improving the general socioeconomic status of women would ultimately increase use of modern contraceptives among women in sub-Saharan Africa. However, while improving the overall socioeconomic status of women is important and should be prioritised, such intervention would only produce a long-term result and requires an unrealistic amount of public expenditure.

More so, despite many studies indicating that high socioeconomic status is associated with contraceptive use, there are studies that suggest that women of low socioeconomic status are more likely to use modern contraception compared to women of high socioeconomic status in sub-Saharan Africa [9, 10]. Findings on the relationship between socioeconomic status and use of modern contraception are mixed. Thus, socioeconomic status might not be the most important determinant of contraceptive use, especially in medically underserved settings of sub-Saharan Africa. Medically underserved setting is defined in this study as areas identified to have huge shortage of health facilities and skewed distribution of available health facilities. In many settings, women residing in urban areas are more likely to use modern contraception compared to women residing in rural areas [11, 12]. What seems peculiar to urban settings in most sub-Saharan Africa countries is better access to health care facilities as compared to rural settings [13]. This suggests that access to health facilities might be the most important determinant of use of modern contraceptives.

The central proposition of this study is that access to health care facility may be the most important determinant of use of modern contraceptive, and as such, efforts to increase modern contraceptive uptake in medically underserved settings should focus on increasing access to health care facilities. Our argument is premised on the consensus among scholars that integration of FP services into maternal and child health and HIV-related programmes is crucial for expanding access to contraceptive, preventing maternal deaths and HIV and improving the reproductive health of women in general [14, 15]. Also, health care visits during antenatal, childbirth, postnatal and child immunisation provide numerous platforms to counsel women about FP and provide FP services [16]. Thus, FP services have become an essential component of reproductive health programmes in many sub-Saharan African countries, especially Nigeria. In Nigeria for instance, the National Reproductive Health Strategic Framework and Plan 2002–2006 recognises FP services and counselling as an effective safe motherhood intervention [17]. Modern contraceptives are available for free in most government-owned health facilities. Also, the evidence of increase uptake of FP services following integration of FP services into maternal and child health and HIV programmes [14, 15] supports our proposition. In other words, there is evidence that postpartum visit attendance increases the use of modern contraceptives [18]. Skilled health workers (midwives and doctors) are the main source of contraceptive information in sub-Saharan Africa [19]. Nevertheless, Haberlen et al. [20] caution that improving access to FP services through integration was not always sufficient to increase the use of effective modern contraception.

However, despite numerous studies on the use of FP methods in sub-Saharan Africa, little is known about how health care visits influence the use of modern contraceptives in medically underserved settings in Nigeria. This indicates a need to examine how access to maternal health services correlate with the use of modern contraception. Drawing from a survey conducted among women who gave birth between 2010 and 2015 in Nasarawa State, Nigeria—a medically underserved state—this paper examined the association between health care visits and contraceptive use as well as explored reasons for non-use of any FP methods.

## Methods

### Study settings

The study took place in Nasarawa State—an understudied state in north central region of Nigeria. Nasarawa State has a very diverse population and suboptimal access to maternal health services. According to Nasarawa State Ministry of Health [21], the population is estimated to be over two million people. Nasarawa State has some peculiar health challenges, which is a synopsis of what is obtainable in other northern parts of Nigeria. The major health challenges facing Nasarawa State are a paucity of skilled human resource for health, inadequate funding, skewed distribution of health facilities, poor infrastructural facilities, low awareness, low community participation, poor access and low utilisation of services among the people. The state set a goal of ensuring that 50% of the population in the state is within 30-min walk or 5 km of a health service facility by the end of 2012, but this goal was not achieved as at 2016.

### Sampling design, participants and sampling

This population-based survey involved 411 participants, selected from 411 households using a two-stage cluster sampling technique. The sample size of 411 was determined through the use of sample size estimation calculator [22]. The calculation was based on a confidence interval of ± 5 and using an infinite population. The sample size estimation was adjusted for possible missing responses. The 2006 census list of enumeration areas (EAs) was the sample frame used for sample selection. According to the NDHS, 25 EAs are needed per state to achieve a representative sample. In the present study, 27 EAs were randomly selected after clustering the EAs into rural, peri-urban and urban. On average, 15 households were randomly selected in each enumeration area. Every 10 household in selected EA was visited to identify study participants until the sample size of 411 women was reached. A participant was eligible if she had delivered in the preceding 5 years of the study. Households without women that gave birth during the specified period or with women who do not want to partake in the study were skipped. The study was conducted between May and September 2016.

### Instrument and measures

The questions used in this study were extracted from a pre-validated questionnaire [23]—used for the national demographic and health survey. Specifically, the DHS questions on reproductive health and family planning were extracted and used to design the study questionnaire. The questionnaire was then piloted among 20 women who were not included in the study. The questionnaire comprises of four key sections. The first part comprises of questions probing participants’ demographic characteristics. Part two of the questionnaire deals with knowledge of various contraceptive methods, while the third part probed the ever use and current use of any form of contraception. The last part examines reasons for non-use of contraception. Questionnaires were administered to the participants through face-to-face interview. Each contraceptive method was described to women before probing if they were aware of each method. Women were subsequently asked if they have used any method of contraceptives to prevent unplanned pregnancy and to state which method they currently use.

### Main outcome variable

The main outcome variable is the use of contraceptive methods. Participants were asked if they have ever used any contraceptive method to prevent pregnancy at the time they do not want to get pregnant and also if they are currently using any contraceptive methods. The responses were dichotomised (yes, no). Women who reported using any modern contraceptive such as condoms, injectables, oral pills, emergency contraception, implants, sterilisation and intrauterine device were classified as “users”. Those who did not indicate any methods were classified as “nonusers”.

### Explanatory variables

The key explanatory variable is health care visits, which was defined as use of skilled birth facilities for antenatal care, child delivery and postnatal care services. Participants were asked if they visited any skilled birth facilities for antenatal care services. Also, participants were asked whether they delivered their last pregnancy in a health facility and whether they receive postnatal care. The responses were dichotomised as “yes” or “no”.

Another key variable of interest is “access to health facility”, which was operationalised as availability of health care facilities in community of residence.

Socio-demographic factors included age (≤ 20 years, 21–25 years, 26–30 years, 31–35 years, 36–40 years and above 40 years), level of education (no formal education, primary education, secondary education and tertiary education and higher), place of residence (rural, peri-urban and urban), marital status (currently married, formerly married and never married), socioeconomic status (low, middle and high) and income level (no income, ≤ 20,000 naira and above 20,000 naira).

Knowledge of contraceptive methods: all participants were asked if they knew a method of preventing pregnancy. A list of contraceptive methods was shown and described to participants. They were asked to respond yes or no to methods they are familiar with.

In this study, socioeconomic status was measured with questions on level of education, employment status, income, ownership of mobile phone, regular watching of television, use of bank account and the Internet. Participants’ socioeconomic status was derived by summing up the scores accrued to each participant from their responses to these questions. A total score of 10 is available, and it represents a high socioeconomic status. A score between 0 and 4 is regarded as low socioeconomic status and scores between 5 and 7 are moderate socioeconomic status, while a score between 8 and 10 is high socioeconomic status.

### Ethical consideration

The University of Fort Hare Ethical Review Committee (AKP031SAJA01) and Ondo State Ministry of Health Ethical Review committee (NHREC/18/08/2016) approved the study protocol. In every community visited, permission to conduct the study was requested from and granted by the community leaders. All participants signed a written informed consent to indicate their voluntary participation in the study. Right to privacy and confidentiality of all participants was protected during and after the data collection.

### Statistical analysis

Descriptive analyses were conducted, and percentages, frequencies and means were reported. To examine factors associated with ever use of contraceptive and modern contraceptive use, cross-tabulation of variables of interest were computed and significant variables were determined with a *p* value less than 0.05. To adjust for potential confounders, significant variables associated with the use of contraceptive methods were included in the binary logistic regression models and odds ratio and 95% confidence intervals were calculated. Logistic regressions were fitted in three stages in order to explore the influence of health care visits and access to health facility in resident community on ever use of contraceptives and modern contraceptive use. First, the effect of each covariate was assessed using univariate models with one independent variable at a time in model 1. The covariates in model 1 were health care visits for antenatal care, child delivery and postnatal care while model 2 combined health care visit variables with access to health facility in resident community. Model 3 consisted of all variables in order to adjust for confounding variables and determine the net effect of the key explanatory variables. However, “place of delivery and postnatal care” were removed from model 3 because they are multi-collinear with “visited health facility for antenatal care”. Likewise, availability of health facility is collinear with place of residence, thus was dropped from model 3. Analysis was conducted using statistical package for social sciences (version 24). To account for complex sampling strategy, weighting factors were applied at various level of analysis. Also, the statistics for complex sample analysis feature of SPSS was used in performing the data analysis.

## Results

### Participants

The average age of the study participants was 28.5 years (SD ± 6.2). The median number of children among the participants was three, with 13 children being the highest number of children given birth to by a woman. Most participants were married (95.1%), Christian (64.0%), own a mobile phone (79.2%) and often watched television (83.2%) (Table 1).

**Table 1:** Demographic characteristics of study participants

Variables	Frequencies	Percentages
Age		
20 years and below	41	10.0
21–25	113	27.5
26–30	124	30.2
31–35	78	19.0
36–40	44	10.7
Above 40	11	2.7
Level of education		
No formal education	85	20.8
Primary education	93	22.7
Secondary education	142	34.7
Tertiary education	89	21.8
Place of residence		
Urban	77	18.7
Peri-urban	125	30.4
Rural	209	50.9
Religion		
Christian	263	64.0
Muslim	144	35.0
Traditional	4	1.0
Marital status		
Currently married	391	95.1
Formerly married	7	1.7
Never married	13	3.2
Employed	167	42.7
Own a phone	326	79.3
Watch television	342	83.2
Own bank account	139	33.8
Use Internet	81	19.7
Number of children		
One	104	25.3
Two	94	22.9
Three	87	21.2
Four	64	15.6
Above four	62	15.0
Income categories		
No income	223	57.5
N20000 and below	138	35.6
Above N20000	27	7.0
Socioeconomic status		
Low	150	38.8
Middle	155	40.1
High	82	21.2
Health care visit for antenatal care		
Yes	373	90.8
No	38	9.2
Health care visit for child delivery		
Yes	239	58.2
No	172	41.8
Availability of health facility in community of residence		
Yes	248	60.3
No	163	39.7

### Health care visits and access to health facility in resident community

The analysis reveals that 90.8% of women visited health centres for antenatal care, 67.9% for child delivery and postnatal care. 60.3% of women resided in communities where there are skilled birth facilities.

### Knowledge of contraceptive methods

Male condom (83.2%) and injectables (85.7%) were the most widely known contraceptive methods among the study participants. Knowledge of FP methods was not universal. Knowledge of traditional FP methods such as lactation amenorrhea (31.6), withdrawal method (57.4) and rhythm method (37.7) was particularly low among study participants. Some participants mentioned the use of cooked leaf/alcohol (*n* = 5), ring from Malam (*n* = 3), potash (*n* = 2), local beads made by herbalist (*n* = 1), soft drinks (Coke, Schweppes and Sprite) (*n* = 4), soap (*n* = 2), vaginal cream (*n* = 1), after sex douching (*n* = 1), Andrew liver salt 6 (8.2%) and salt and water (*n* = 48) as methods of preventing unintended pregnancy.

As shown in Table 2, knowledge of contraceptive methods was significantly lower among women who reside in rural areas.

**Table 2:** Knowledge of contraceptive methods

Types of contraceptives	All (*N* = 411)	Residence		*p* value
		Urban (*N* = 202)	Rural (*N* = 209)	
Male sterilisation	211 (51.3)	123 (60.9)	88 (42.1)	< 0.001
Female sterilisation	311 (75.7)	175 (86.6)	136 (65.1)	< 0.001
IUD	232 (56.4)	128 (63.4)	104 (49.8)	< 0.05
Injectable	317 (85.7)	181 (89.6)	136 (65.1)	< 0.001
Implants	311 (75.7)	175 (86.6)	136 (65.1)	< 0.001
Oral pills	328 (79.8)	189 (93.6)	139 (66.5)	< 0.001
Male condom	342 (83.2)	192 (95.0)	150 (71.8)	< 0.001
Female condom	294 (71.5)	176 87.1)	118 (56.5)	< 0.001
Emergency contraception	196 (47.7)	115 (56.9)	81 (38.8)	< 0.001
Standard day method	242 (58.9)	151 (74.8)	91 (43.5)	< 0.001
Lactation amenorrhea	130 (31.6)	98 (48.5)	32 (15.3)	< 0.001
Rhythm method	155 (37.7)	123 (60.9)	32 (15.3)	< 0.001
Withdrawal method	236 (57.4)	161 (79.7)	75 (35.9)	< 0.001
Folks methods	73 (17.8)	54 (26.7)	10 (4.8)	< 0.001

Note: Folks’ methods include cooked leaf/alcohol, ring from Malam, potash, local beads made by herbalist, Coke, Schweppes, Sprite, soap, vaginal cream, after sex douching, and Andrew liver salt and salt and water

### Contraceptive prevalence rate

About 10% of the study participants (*n* = 39) did not respond to the question on the use of contraceptives to prevent unintended pregnancy. Over half of the women (55.4%; *n* = 206/372) reported ever use of any contraceptive methods. Half of the women (*n* = 189/372) reported current use of any contraceptive methods. The prevalence of modern contraceptive use was 46% (*n* = 171/272). Condom is the most used contraceptive method and was reported by more than one in three women currently practicing any form of contraception. One in five women reported current use of injectables, 17.5% pills and 15.9% implants. The use of traditional FP methods was very low (6.9%). A few women used folk methods (2.6%).

### Factors associated with use of family planning methods

Age, place of residence, level of education, religion, ownership of mobile phone and bank account, access to TV and Internet, marital status, health care visits during antenatal care and childbirth and availability of health care facility in the community of residence were significantly associated with ever use of any FP method. Similarly, age, place of residence, level of education, religion, ownership of mobile phone and bank account, access to TV and Internet, marital status, health care visits during antenatal care and childbirth and availability of health care facility in the community of residence were significantly associated with the use of any modern FP method (Table 3).

**Table 3:** Chi-square statistics showing factors association with ever use of any family planning methods and any modern methods

Variable	Ever used any methods (*n* = 206)	*p* value	Any modern method (*n* = 189)	*p* value
Age				
20 and below (*n* = 37)	9 (24.3)	< 0.05	7 (18.9)	< 0.05
21–25 (*n* = 102)	56 (54.9)		50 (49.0)	
26–30 (*n* = 113)	68 (60.2)		64 (56.6)	
31–35 (*n* = 72)	47 (65.3)		43 (59.7)	
36–40 (*n* = 37)	20 (54.1)		20 (54.1)	
Above 40 (*n* = 11)	6 (54.5)		5 (45.5)	
Parity				
One child (*n* = 93)	43 (46.2)	> 0.05	41 (44.1)	> 0.5
Two children (*n* = 83)	45 (54.2)		44 (53.0)	
More than two children (*n* = 196)	118 (60.2)		104 (53.1)	
Place of residence				
Urban (*n* = 71)	53 (74.6)	< 0.001	51 (71.8)	< 0.001
Peri-urban (*n* = 110)	84 (76.4)		80 (72.7)	
Rural (*n* = 191)	69 (36.1)		58 (30.4)	
Level of education				
No schooling (*n* = 75)	25 (33.3)	< 0.001	19 (25.3)	< 0.001
Primary (*n* = 86)	47 (56.0)		44 (51.2)	
Secondary (*n* = 131)	75 (57.3)		71 (54.2)	
Higher degree (*n* = 80)	58 (72.5)		55 (68.8)	
Religion				
Christianity (*n* = 236)	144 (61.0)	< 0.05	135 (57.2)	< 0.05
Islam (*n* = 136)	62 (45.6)		54 (39.7)	
Mobile phone ownership				
Yes (*n* = 296)	182 (61.5)	< 0.001	166 (56.1)	< 0.001
No (*n* = 76)	24 (31.6)		23 (30.3)	
Access to TV				
Yes (*n* = 310)	196 (63.2)	< 0.001	180 (58.12)	< 0.001
No (*n* = 62)	10 (16.1)		9 (14.5)	
Owned bank account				
Yes (*n* = 122)	83 (68.0)	< 0.001	80 (65.6)	< 0.001
No (*n* = 250)	123 (49.2)		109 (43.6)	
Access to Internet				
Yes (*n* = 69)	49 (71.0)	< 0.05	48 (69.6)	> 0.001
No (*n* = 303)	157 (51.8)		141 (46.5)	
Marital status				
Currently married (*n* = 354)	201 (56.8)	< 0.05	186 (52.5)	< 0.05
Formerly married (*n* = 6)	3 (50.0)		1 (16.7)	
Never married (*n* = 12)	2 (16.7)		2 (16.7)	
Measure of social status				
Low SES (*n* = 73)	60 (43.8)	< 0.001	50 (36.5)	< 0.001
Middle SES (*n* = 141)	88 (62.4)		83 (58.9)	
High SES (*n* = 137)	50 (68.5)		48 (65.8)	
Health care visit for antenatal care				
Yes (*n* = 338)	198 (58.6)	< 0.001	183 (54.1)	< 0.001
No (*n* = 34)	8 (23.5)		6 (17.6)	
Place of ANC				
Tertiary, secondary and private (*n* = 179)	126 (70.4)	< 0.001	118 (65.9)	< 0.001
PHC (*n* = 159)	72 (45.3)		65 (40.9)	
Home (*n* = 34)	8 (23.5)		6 (17.6)	
Who assisted during childbirth				
Skilled health worker (*n* = 254)	155 (61.0)	< 0.001	145 (57.1)	< 0.001
Unskilled attendants (*n* = 118)	51 (43.2)		44 (37.3)	
Health care visit for childbirth				
Yes (tertiary, secondary and private) (*n* = 144)	104 (72.2)	< 0.001	100 (69.4)	< 0.001
PHC (*n* = 70)	31 (44.3)		28 (40.0)	
Not in health facility (*n* = 158)	71 (44.9)		61 (38.6)	
Availability of health facility in community of residence				
Yes (*n* = 225)	151 (67.1)	< 0.001	145 (64.4)	< 0.001
No (*n* = 147)	55 (37.4)		44 (29.9)	

*SES* socioeconomic status

### Health care visits and use of contraceptive methods

Model 1 presented in Tables 4 and 5 is the unadjusted binary regression showing independent contribution of health care visits during antenatal care, childbirth and postnatal care to use of contraceptives. Health care visits during antenatal care, childbirth and postnatal care were independent predictors of contraceptive use. Women who visited health facilities for antenatal care were more than four times more likely to have ever used a contraceptive method or currently use a modern contraceptive method compared to women who do not. Women who received postnatal care were over two times more likely to have ever used any FP method or report current use of modern contraception compared to women who do not, likewise women who gave birth in skilled birth facilities.

**Table 4:** Condensed multiple logistic regression models showing odds for ever use of any contraceptive methods

Variables	Model 1 UOR (95% CI)	Model 2 AOR (95% CI)	Model 3 AOR (95% CI)
Visited health facility for antenatal care			
Yes	4.5 (2.0–10.5)***	3.1 (1.3–7.5)*	2.4 (1.0–5.8)
No (ref)	1	1	1
Place of child delivery	2.1 (1.4–3.2)***	1.3 (0.6–2.7)	N/A
Health facility			
Home (ref)			
Received postnatal care			
Yes	2.3 (1.5–3.5)***	1.2 (0.6–2.7)	N/A
No (ref)	1	1	
Availability of health facility in community			
Yes		3.1 (1.8–5.2)***	N/A
No (ref)		1	
Place of residence			
Urban			5.1 (2.7–9.5)**
Peri-urban			4.5 (2.3–8.8)**
Rural (ref)			1
Age			
> 20 years			3.3 (1.4–7.7)*
≤ 20 years (ref)			1
Level of education			
Higher degree			2.0 (1.01–4.1)*
Secondary			1.07 (0.5–2.2)
Primary			1.2 (0.5–2.9)
No schooling (ref)			1

Listwise deletion was used to remove 39 cases of missing responses in the model

Model 1 unadjusted model examining independent maternal health care visits predictors of ever use of any contraception

Model 2 adjusted for demographic factors

Model 3 adjusted for place of childbirth, postnatal care and availability of health facility in community (these variables are possible confounders)

*AOR* adjusted odds ratio, *UOR* unadjusted odds ratio, *ref* reference, *N/A* not included in the model

***Statistically significant (*p* < 0.001), *statistically significant (< 0.05)

**Table 5:** Condensed multiple logistic regression models showing odds for current use of modern contraceptives

Variables	Model 1 UOR (95% CI)	Model 2 AOR (95% CI)	Model 3 AOR (95% CI)
Visited health facility for antenatal care			
Yes	5.6 (2.1–14.8)***	3.3 (1.2–9.4)*	3.2 (1.1–8.8)*
No (ref)	1	1	1
Place of child delivery			
Health facility	2.3 (1.5–3.6)***	0.7 (0.3–1.6)	N/A
Home (ref)			
Received postnatal care			
Yes	2.8 (1.8–4.5)***	1.7 (0.8–3.8)	N/A
No (ref)	1	1	1
Availability of health facility in community of residence			
Yes		2.8 (1.7–4.9)***	1.1 (0.5–2.4)
No (ref)		1	1
Place of residence			
Urban			3.5 (1.6–7.8)**
Peri-urban			3.4 (1.6–7.2)**
Rural (ref)			1
Age			
> 20			3.6 (1.4–9.3)*
≤ 20			1
Level of education			
Higher degree			2.5 (1.2–5.1)*
Secondary			1.3 (0.6–2.8)
Primary			1.7 (0.7–3.8)
No schooling			1

Listwise deletion was used to remove 39 cases of missing responses in the model

Model 1 unadjusted model examining independent association of maternal health visits and modern contraceptive use

Model 2 adjusted for demographic factors

Model 3 confounding variables adjusted for were postnatal care and place of child delivery

*AOR* adjusted odds ratio, *UOR* unadjusted odds ratio, *ref* reference, *N/A* not included in the model

***Statistically significant (*p* < 0.001), *statistically significant (< 0.05)

Model 2 examined the net effect of health care visits for antenatal, childbirth and postnatal care adjusted for availability of health facility in the community of residence. The analysis reveals that only health care visit for antenatal care and access to health facility in resident community were independent predictors of contraceptive use. Women who resided in communities where there were health facilities were about three times more likely to report ever use of any FP methods or currently using any modern FP methods compared to women who do not (Tables 4 and 5).

In model 3, after adjusting for confounding factors, health care visit for antenatal care remains an independent determinant of modern contraceptive use along with place of residence, level of education and age. Women who visited health facilities for antenatal care were more than three times more likely to report current use of any modern contraceptive method compared to women who did not visit any skilled birth facilities. However, health care visit for antenatal care did not predict ever use of contraceptive in the adjusted model.

Women who reside in urban and peri-urban areas were over four to six times more likely to have ever used any FP method compared to women who reside in rural areas. Likewise, women who reside in urban areas were over three times more likely to report current use of any modern contraceptive method compared to women who reside in rural areas. Women aged above 20 years were more than three times more likely to report ever used a FP method or currently using a modern contraceptive method. Women who had higher education were more than two times more likely to report ever used a FP method or currently using a modern contraceptive method.

### Reasons for non-use of contraceptive

Of women who are not using any form of contraceptive (*n* = 144), lack of knowledge (23.6%), negative perception of contraceptive side effects (17.4%) and lack of interest (21.5%) were the commonly stated reasons for not using any FP method. 18.1% of the women did not use contraceptive because they were expecting to become pregnant. Only a few women stated they could not afford it (5.6%), lack of regular sex (5.6%) and husbands’ refusal (1.4%).

## Discussion

This study examined the relationship between health care visits (during antenatal, childbirth and postnatal care) and use of contraceptive methods among parous women in a medically underserved setting of Nigeria. The study found that use of modern contraceptives was significantly associated with health care visits. Women who utilised skilled birth facilities for antenatal care and postnatal care were significantly more likely to use any modern contraceptive after controlling for age and level of education. The findings of the current study corroborate our assumption that access to health facilities is the main determinant of contraceptive use. This is so considering that FP services have been integrated to reproductive health services in Nigeria. Also, health care visits during pregnancy, postnatal care and child immunisation enable health workers to counsel women on the importance of child spacing. In addition, the integration of FP services into maternal and child health services provides a unique opportunity to provide FP information and services to women [14, 15]. The present study corroborates Akinlo et al. [24] and Masho et al. [18] who claim health care visit increases the use of modern contraceptives. In the present study, women who visit skilled facilities for antenatal care were over three times more likely to use modern contraceptive methods compared to women who do not. Masho et al. [18] found that women who attend postpartum care visit were 50% more likely to use modern contraceptive methods compared to women who do not. Our finding highlights the significance of health care visits for antenatal care for the uptake of modern FP methods. In all the three models, health care visit during antennal care remains a significant determinant of contraceptive use. Thus, a priority towards improving the use of modern FP methods in medically underserved settings would entail expanding access to health care facilities.

Even though high level of knowledge of FP was reported in this study, women who did not visit a skilled facility during pregnancy were the least likely to have knowledge of contraceptive methods. This finding further underscores the importance of health care visits as the main pathways to learn about mechanism and importance of contraceptives. Many studies have shown that health workers are the primary source of contraceptive information [19, 25]. Thus, it is unsurprising that lack of knowledge of FP was the most stated reason for non-use of any FP methods in our study settings unlike fear of side effects of contraceptives in other studies [26–28].

The study also examined the relationship between access to health facility in resident community and use of contraceptive methods. We found that women who reside in communities where there are health facilities were over two times more likely to use contraceptives compared to women who do not. However, access to health facility in resident community was a confounding factor in model 3. Generally, women who reside in rural medically underserved settings were the least likely to use contraceptives, which is consistent with the literature [29]. Our study shows that women who reside in urban areas were over six times more likely to have ever used any FP method compared to women who reside in rural areas. Likewise, women who reside in urban areas were over three times more likely to report current use of any modern contraceptive methods compared to women who reside in rural areas. Clearly, there is geographical inequality in the use of FP methods in the study settings. One possible explanation for the result is the lack of access to health facilities in many rural communities in our study settings. A study reports that women living at least 5 km from a health facility were less likely to use contraception [30]. There is evidence showing that lack of availability of contraceptives is the main reason for non-use [31]. Our study shows that the rate of use of any family planning methods is higher in communities where there are health care facilities compared to those without any health care facility. It is therefore imperative to increase access to health care facilities in medically underserved settings in order to achieve the Sustainable Development Goal target of universal access to sexual and reproductive health services including family planning by 2030.

### Study limitations

One limitation of our study is the use of self-reporting to elicit information on contraceptive use. Considering the sensitive nature of the topic in our study setting, contraceptives prevalence could have been underestimated. However, the use of face-to-face interview enables the researchers to probe in order to understand how women prevent unplanned pregnancy during birth intervals. Also, due to the cross-sectional design of this study, a causal association between health care visit and use of modern contraception cannot be established. In addition, women were asked to reflect on events (use of maternal health services), which take place over a period of 1 to 5 years. This methodology, even though feasible, is subjected to recall bias. Our measure of socioeconomic status did not account for spousal’s socioeconomic status, which might be an important determinant of use of maternal health services and contraceptives. Nonetheless, our study is among the few studies to examine the effect of maternal health services utilisation on contraceptive use.

## Conclusion

Our findings suggest that health care visits are a key determinant of use of family planning methods and also underscore the importance of health care visit to the uptake of FP methods. Expanding access to health facilities in medically underserved settings is greatly needed in order to achieve the Sustainable Development Goal target of universal access to sexual and reproductive health services including family planning by 2030.

## Abbreviations

FP: Family planning
IUD: Intrauterine device
